# Comparative Clinical Performances of Tunneled Central Venous Catheters versus Arterio-Venous Accesses in Patients Receiving High-Volume Hemodiafiltration: The Case for High-Flow DualCath, a Tunneled Two-Single-Lumen Silicone Catheter

**DOI:** 10.3390/jcm12144732

**Published:** 2023-07-17

**Authors:** Bernard Canaud, H. Leray-Moragues, Leila Chenine, Marion Morena, George Miller, Ludovic Canaud, Jean Paul Cristol

**Affiliations:** 1 School of Medicine, Montpellier University, 34090 Montpellier, France; 2 MTX Consulting International, Rue des Carmelites, 34090 Montpellier, France; 3 AIDER-Santé, CHARLES, Mion Foundation, 34000 Montpellier, France; helene.moragues@gmail.com (H.L.-M.); jp-cristol@chu-montpellier.fr (J.P.C.); 4 Nephrology, Intensive Care, Dialysis & Transplantation, Lapeyronie University Hospital, 34090 Montpellier, France; 5 PhyMedExp, Department of Biochemistry and Hormonology, INSERM, CNRS, University Hospital Center of Montpellier, University of Montpellier, 34000 Montpellier, France; m-morenacarrere@chu-montpellier.fr; 6 Medical Components, Inc., Clinical Affairs, Harleysville, PA 19438, USA; 7 Chest and Vascular Surgery Department, CHU Montpellier, 34000 Montpellier, France

**Keywords:** vascular access, tunneled central venous catheter, dialysis clinical performances, diffusive dialysis dose, convective dialysis dose, effective blood flow, recirculation

## Abstract

Tunneled central venous catheters (CVC) are mainly considered as a rescue vascular access option in dialysis but are still used on approximately one quarter of prevalent patients worldwide even though they are associated with poor performances and higher risks. Study design: in this retrospective single-center study, we aimed to report on the clinical performances achieved with high-flow tunneled CVCs (DualCath or DCath) and compared them with arteriovenous accesses (AVAs, e.g., AV fistula, AV graft, and Thomas Shunt) in a hospital-based dialysis unit. Methods: Sixty-eight stage 5 chronic kidney disease dialysis-dependent patients (CKD5D) receiving high volume hemodiafiltration were followed-up with for 30 months. The study consisted of two phases: baseline cross-sectional and longitudinal follow-ups of key performance indicators. Clinical performances consisting of effective blood flow and blood volume, recirculation, urea and ionic Kt/V, total Kt, ultrafiltration volume, and percent reduction in β2-M were measured monthly as part of quality control in our unit. Results: At baseline, the effective blood flow using a DCath was close to 400 mL/min, similar to an AVA. Recirculation with a DCath (7%, 6–13%) was higher than with an AVA. The diffusive dialysis dose delivered with a DCath (spKt and eKt/V) and convective dialysis dose achieved with a DCath were slightly lower than those achieved with AVAs, but they were still much higher than is recommended by guidelines. The percent reduction in β2-M achieved with a DCath was also 4 to 10% lower than that achieved with an AVA. On longitudinal follow-up, the main clinical performance indicators of DCaths (total Kt and total ultrafiltration volume, L/session) were maintained as very stable over time and close to those achieved with AVAs. Conclusions: As shown in this study, high-flow DualCath tunneled two-single-lumen silicone catheters may be used to deliver high volume hemodiafiltration in a reliable and consistent manner without compromising clinical performance. These results relied on the specific design of the two silicone cannulas and the strict adherence to best catheter practices.

## 1. Introduction

Tunneled central venous catheters (CVC) are considered as a rescue vascular access option in dialysis patients by almost all clinical practice guidelines, and they are associated with higher morbidity, lower performances, and greater cost [1,2,3,4]. However, the prevalent use of CVCs in dialysis remains high, ranging almost from zero up to 50%, with a median value of 25% of dialysis patients using these catheters worldwide [5,6,7,8]. These facts tend to support the notion that CVCs are still needed in the vascular access care offerings [9,10,11] for kidney disease management since they provide an interesting option to ensure care continuity in CKD patients facing recurring vascular access problems that were well recognized in their last KDOQI issue [2].

The intrinsic limitations of CVCs are acknowledged and analyzed in various reports [12,13]. These limitations relate to the geometry of the cannulas (internal lumen diameter, length, and polymer characteristics), the catheter design (single- or dual-lumen design and central and side holes), or the catheter tip’s location within the venous central system (superior vena cava or right atrium) [14,15]. In addition, tunneled CVCs bear higher risks (dysfunction, infection, and thrombosis) that can be mitigated by better handling and locking solutions [16,17,18,19]. These facts have raised several controversies in the scientific literature, with reported pros and cons [11,20,21,22]. It was not our intent to enter this polemic; rather, we intended to assess the clinical performances delivered by means of tunneled CVCs compared to arterio-venous accesses (AVA) in stage 5 chronic kidney disease (CKD5D) patients receiving high-volume hemodiafiltration as a mainstream treatment.

In this study, we aimed to report on the clinical performances achieved with high-flow tunneled CVCs (DualCath or DCath) and compared them with the various AVAs used at the same time in our hospital-based dialysis unit.

## 2. Material and Methods

### 2.1. Study Design

This was a retrospective single-center study conducted at Lapeyronie Hospital in Montpellier, France. The study utilized data extracted from electronic dialysis medical records of patients in the teaching hospital-based dialysis unit. The data collection period spanned from 1 October 2009 to 31 May 2012, with a follow-up period of 30 months.

The study consisted of two parts: a baseline assessment of clinical performances using data from three consecutive months, categorizing patients based by vascular access type (tunneled CVCs and AVAs), and a longitudinal follow-up of clinical performances up to 30 months, also considering vascular access type.

The clinical dialysis performance indicators included effective blood flow, access recirculation, urea spKt/V and dpKt/V (Daugirdas), ionic dialysance measurement (online clearance measurement (OCM)), total clearance delivered per session (Kt), total ultrafiltration volume (VUF), substitution volume (VSUB) per session, albumin, and hemoglobin.

### 2.2. Patients

This study enrolled 68 stage 5 chronic kidney disease dialysis-dependent patients (CKD5D) receiving high-volume hemodiafiltration. Ten were incident patients (<3 months of treatment) and 58 were prevalent patients (≥3 of months treatment). Some incident patients were converted during the study to arteriovenous fistulas or graft accesses.

### 2.3. Ethics Statement

The study was conducted according to the principles of the Declaration of Helsinki and in compliance with the International Conference on Harmonization/Good Clinical Practice regulations. In accordance with French Law, the study was registered at “Ministère de la Santé et des Solidarités” after approval by the Montpellier University Hospital’s ethics committee (Comité de Protection des Personnes Sud Méditerranée IV) with the following number: DC-2008-417. All patients provided their written informed consent.

### 2.4. Vascular Accesses

The DualCaths (DCaths) were manufactured by MedComp in the United States of America and distributed in Europe [23,24,25,26]. A DCath is a medical device composed of two separate single-lumen silicon catheters and their respective tubing extension adaptors. The intravascular cannula is made of radiopaque silicone polymer tubing, measuring 40 cm in length, with inner and outer diameters of 2.0 mm and 3.2 mm, respectively. The lengths of the cannulas are adjusted during insertion to accommodate a patient’s body size. Consequently, DCaths may vary in length depending on a patient’s height and the side of insertion (25–27 cm for the right side and 30–32 cm for the left side). At the distal tips of the catheters, there are six spiral holes. The tubing extension adaptors are made of a silicone polymer that measures 6–7 cm in length and terminates in a nylon Luer lock connection device. The connection device is color-coded, with blue indicating venous use and red indicating arterial use. Venous fistulas (VFs) are primarily native fistulas located in the forearm while arteriovenous grafts (AVGs) are constructed using polytetrafluoroethylene (PTFE) and placed as a loop on the arm. Thomas shunts, on the other hand, are inserted into the iliac and femoral vasculature.

#### 2.4.1. DualCath Looking and Imaging (Figure 1)

**Figure 1:**
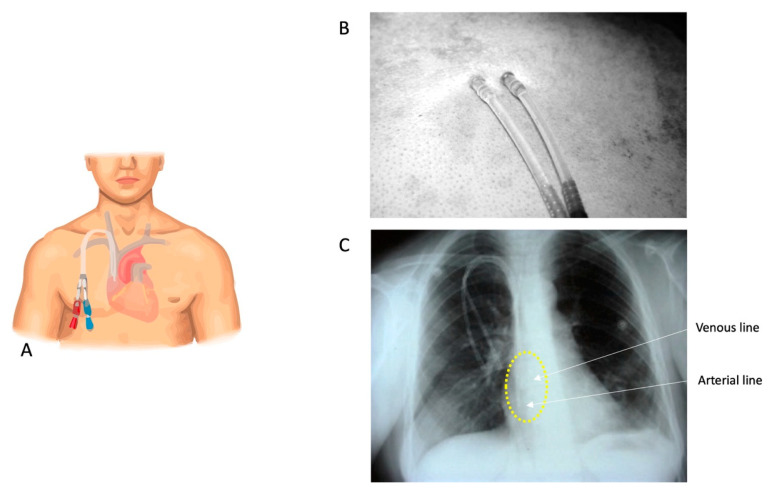
(**A**) A stylized cartoon representation of a DCath inserted into the right internal jugular vein. (**B**) A DCath’s skin exit site. (**C**) A chest X-ray conducted before catheter use, serving the purpose of confirming the catheter’s position and identifying any abnormalities or complications. In this X-ray, the positions of the catheter tips are highlighted with a yellow circle, indicating that they are located at the junction of the superior vena cava and the right atrium. Additionally, an arrow illustrates the arterial and venous tips of the catheter. According to the annotation, the two tips are approximately 2 cm apart from each other.

DCath insertion is a procedure performed by a nephrologist under local anesthesia. It involves inserting two silastic cannulas into a patient’s chosen central vein, primarily the right internal jugular vein [27]. The cannulas are tunneled under the skin, shortened, and secured with sutures and a silicone rubber collar, as shown in Figure 1A,B. After rinsing and clamping, the cannulas are anchored together with sutures. The skin incision is closed and the cannulas are rinsed, locked, and capped. The procedure requires strict aseptic conditions, and it may sometimes require general anesthesia for complex cases or anxious patients.

#### 2.4.2. DualCath and Vascular Access Management and Handling

DCaths are solely used for hemodialysis and strictly prohibited for other purposes. Trained nurses handle DCaths with sterile precautions, wearing gowns, masks, hats, and gloves. During connection, the remaining blood material is removed and flushed with saline. The extracorporeal blood circuit is initiated, and during disconnection, blood is flushed back to the patient. Each DCath line is filled with a mixture of saline and citric acid solution, capped with a Luer lock cap. The DCath is then wrapped in sterile gauze and covered with a waterproof dressing until the next dialysis session. 

#### 2.4.3. Arteriovenous Access Management

Arteriovenous fistulas (AVFs) or grafts (AVGs) and Thomas shunts are handled with particular care and strictly hygienic conditions that rely on betadine as a skin disinfectant and use sterile materials, drapes, and gloves, as well as masks, gowns, and caps, for the operators at the time of access handling. The needling is performed by trained nurses using the laden rope method with fistula needles of 15 gauges in a majority of patients.

### 2.5. Clinical Performances Assessed

The baseline clinical performances consisted of six key performance indicators that included the effective blood flow (mL/min), venous pressure (mmHg), vascular access recirculation (VA.REC) (%), single pool Kt/V, double pool Kt/V, and convection volume (L/session).

These indicators were measured in the six following vascular accesses: native arteriovenous fistula (AVF), arteriovenous graft (PTFE) (AVG), right femoral DCath (RFDC), right internal jugular Dcath (RIJDC), left internal jugular Dcath (LIJDC), and Thomas shunt.

In addition, cumulative clinical performances comparing DCaths and grouped AVAs over a 30-month follow-up period were performed.

#### 2.5.1. Blood Flow (QB, mL/min)

Blood flow measurements were performed by means of bubble transit time on 1 m of a calibrated segment of the arterial line [28]. This method is currently used in the study unit and fits perfectly with the use of Transonic data. The calculation of total effective blood flow relied on following set out in 2.5.2.

#### 2.5.2. Total Blood Volume Processed (TBVP, L/Session)

The total blood volume processed was calculated from the dialysis machines as the product of the effective blood flow (QB) times the treatment time (tHD), as follows: TBVP = QB × tHD.

#### 2.5.3. Vascular Access Recirculation (VA.REC, %)

The total recirculation (T.REC) was calculated by using the urea dosing issued from three blood samplings (artery (ART) and venous (VEN) sampling, followed by a second artery sampling 2 min after the blood had stopped being pumped (ART_+2_)), as follows [28,29]:

T. REC = (ART_+2_ − ART)/(ART_+2_ − VEN) × 100.

This method was validated in the study unit and has been used as standard of care for years. The results fit perfectly with the recirculation obtained using the blood thermal monitoring device, which also assessed total recirculation. When compared to the Transonic data, it was shown that 10% came from the cardiopulmonary bypass created by the arteriovenous bypass [16,30]. A 10% value of cardiopulmonary bypass was then subtracted from arteriovenous access measurements—but not from the tunneled central venous accesses—as follows:

VA. REC = T.REC − 10 (for arteriovenous accesses only).

#### 2.5.4. Dialysis Dose Delivery

The dialysis dose delivery was assessed with three key indicators: firstly, the urea clearance expressed as fractional urea clearance (i.e., _urea_Kt/V) or its surrogate using ionic clearance (online clearance measurement, _OCM_Kt/V) [31,32] and as total urea clearance (Kt) delivered per session (i.e., liters per session) to quantify the small molecule clearances [31,33,34]; secondly, the total ultrafiltration volume (liter per session) or convective dialytic dose used as surrogate marker of middle and large molecule clearances [35]; and thirdly, the percent reduction in β2M (%) per session.

##### Urea Kt/V

The urea Kt/V was calculated from the pre- and post-dialysis blood sampling using the single-pool [36] (sp) and equilibrated (e) double-pool Kt/V formula from Daugirdas [37] as set out below. We note that the post-dialysis blood sampling was performed after reducing the blood pump down to 50 mL/min for two minutes.

_sp_Kt/V = −Ln (Urea_post_/Urea_pre_ – 0.008 × t_HD_) + (4 − 3.5 × Urea_post_/Urea_pre_) × WL/BW_post_

_e_Kt/V = _sp_Kt/V − (0.6 × _sp_Kt/V/t_HD_) + 0.03,

where WL is the weight loss during the session, BW the body weight, and t_HD_ is the effective treatment time.

##### Ionic Dialysance and _ocm_ Kt/V

The ionic dialysance Kt/V and _ocm_Kt/V were measured and calculated from the dialysis monitor using integrated ionic dialysance (K_ocm_) performed across dialysis sessions, effective HD machine treatment times (t_HD_) and total body water (V) derived using the Watson formula by default.

##### Total Kt (TKt, L/Session)

The total Kt was calculated as the product of ionic clearance (K_ocm_) and effective treatment time (t_HD_) of the dialysis sessions as TKt = K_ocm_ × t_HD._

##### Total Ultrafiltration Volume (VUF, L/Session)

The total ultrafiltration volume (VUF) is the sum of the online substitution volume infused during an HDF session (VSUB) and the intradialytic weight loss (WL) to correct fluid overload. This indicator is used as a surrogate for the convective dialytic dose delivered per session [35].

##### Percent Reduction in β2-Microglobulin (PRβ2M)

The percent reduction in β2M was used as a marker of HDF efficiency as the patients were receiving HDF treatments [35]. The post-dialysis β2M was corrected for hemoconcentration using the Bergstrom formula [38], as follows:

_cor_ β2M = _post_ β2M (1 + ∆BW/0.2BW_post_),

where ∆BW is the difference between the pre- and post-treatment BW measurements.

The PRβ2M was calculated as follows:

PRβ2M = 1 − (_cor_β2M_post_/β2M_pre_) × 100.

##### Normalized Protein Catabolic Rate (nPCR)

The nPCR (g/kg/24 h) was calculated from the urea kinetic modeling using the three points method [39] (pre/post and before the next dialysis session).

The blood samples were analyzed on the same day they were taken in the central laboratory of the study hospital using validated autoanalyzer methods.

### 2.6. Statistics

Statistical analyses were performed using JASP Software 0.17.1 (University of Amsterdam). Normality and variance equality tests were made for all data, and the statistical significance level was set to *p* < 0.05 for all analyses. Continuous variables were reported as means (SD) or as medians (25–75%) depending on whether the data were normally distributed or not. Categorical variables were reported as percentages with numbers in parentheses. Unpaired normally distributed continuous variables were tested for differences using unpaired t-tests. Unpaired non-normally distributed continuous data were analyzed using Mann–Whitney tests. Pearson’s or Spearman’s tests were used for correlation analyses depending on whether the data were normally distributed or not, respectively.

## 3. Results

### 3.1. Patient Characteristics

The cohort consisted of 27 females (average age of 72 years old (66.5–75.5)) and 41 males (average age of 68.5 years old (60.0–75.0)) with a dialysis vintage of 9 (2.5–30) months. The primary kidney diseases present were as follows: hypertension and/or ischemic nephropathy, 28 (41.2%); diabetic kidney disease, 11 (16.2%); chronic glomerulonephritis, 8 (11.8%); toxic kidney disease, 6 (8.8%); autosomic polycystic kidney disease, 4 (5.9%); Alport syndrome, 1 (1.5%); interstitial chronic nephritis uropathy, 1 (1.5%); and unknown, 9 (13.2%). Six patients with toxic kidney disease had received an organ transplant (one heart, two livers, and three kidneys).

### 3.2. Renal Replacement Treatment Schedule

All patients received online post-dilution hemodiafiltration. The treatment schedule consisted of three sessions weekly, with treatment times of 4 h for 90% of the patients and 3 or 3.5 h for the remaining 10%. The single-use hemodiafilters were made of high-flux polysulfone or polyethersulfone (FX1000, 34.4%; FX800, 59.4%; and Arylane H9, 2%). The anticoagulation process consisted of a single bolus injection of Fraxiparin (Nadroparin 2000–6000 IU), a low molecular weight heparin, in the venous needle or within the catheter line before initiating the extracorporeal circuit.

### 3.3. Baseline Clinical Performances (3 Months)

**Figure 2:**
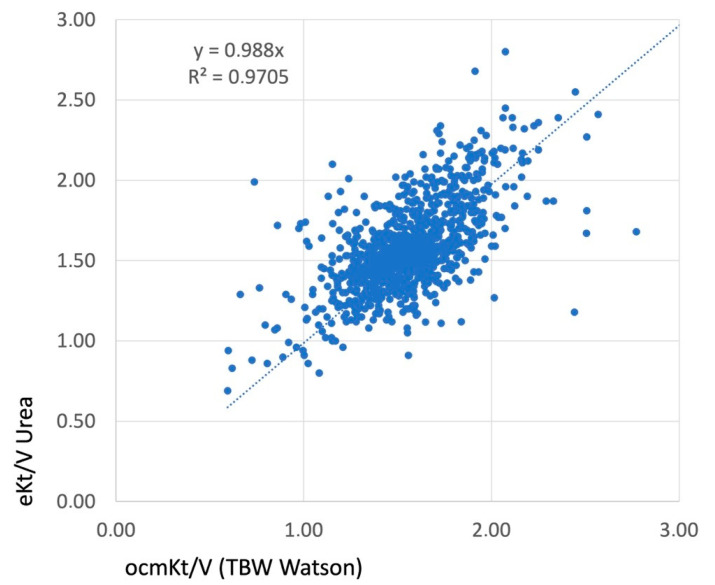
The correlation achieved between the _OCM_Kt/V, measured as an online clearance using ionic dialysance, and _e_Kt/V, calculated as the equilibrated Kt/V using the Daugirdas second generation formula. The volume (V) utilized in this analysis was obtained from the Watson formula. The presence of a linear correlation and a high R2 value substantiated the accuracy and validity of this indicator.

The baseline clinical performances are presented in Table 1. N stands for the number of patients given (first row) and the number of measures given (second row). The median and interquartile values are given for each key performance indicator for each access type. As shown, the effective blood flow achieved was virtually similar (400 mL/min) in each vascular access, including those using DCaths. Recirculation was significantly higher with a DualCath (an average of 7% (6 to 13%)) but higher with an LIJDC. The dialysis dose delivered (Kt/V) was virtually equivalent across all vascular accesses (if we account for the differences in the patients’ body weight and total body water measurements). In all cases, including those using DCaths, the median values of the Kt/V were superior to 1.70 (spKt/V) or 1.5 (eKt/V). We note that we also explored the correlations between the _OCM_Kt/V and eKt/V values to confirm the validity of such routine parameters, as observed in Figure 2. The convective volumes tended to be lower with DCaths compared to the arteriovenous accesses (AVF, AVG, and TS), but they were still higher than 23 L per session, as recommended. The percent reduction in β2M with the use of a DCath was 4 to 10% lower than when using an AVF, an AVG, or a Thomas shunt, though it still achieved 72.6 to 75.4 percent per session.

### 3.4. Clinical Performances (Over a 30-Month Follow-Up Period)

#### 3.4.1. Cumulative Clinical Performances Comparing DualCaths (DCaths) and Grouped Arterio-Venous Accesses (AVAs)

The vascular accesses were grouped as AVAs and DCaths, and the median values of the key performance indicators are presented in Table 2. As shown, the patients bearing DCaths tended to have smaller body weights compared to the patients treated through AVAs. They also tended to have slightly lower blood flows and higher recirculation, translating to a slightly and significantly lower dialysis dose delivered for the small molecules (Kt/V and Kt) but not for the convection volume (22.5 vs. 25.4, NS) and percent reduction in β2M per session (74 vs. 78%, NS). Indeed, the effective dialysis doses achieved with DCaths were much higher than that recommended by best practice guidelines. This fact was confirmed by the stability over time of the nutritional markers such as albumin, prealbumin (transthyretin), or normalized protein catabolic rate (nPCR). The CRP and fibrinogen used as indicators of inflammation were similar in all vascular access groups. Additionally, the key parameter indicators of dialysis adequacy (fluid volume status, blood pressure, electrolytic control, bone mineral disorder, and anemia) were maintained at target levels for both types of vascular access.

#### 3.4.2. Longitudinal Follow-Up

**Figure 3:**
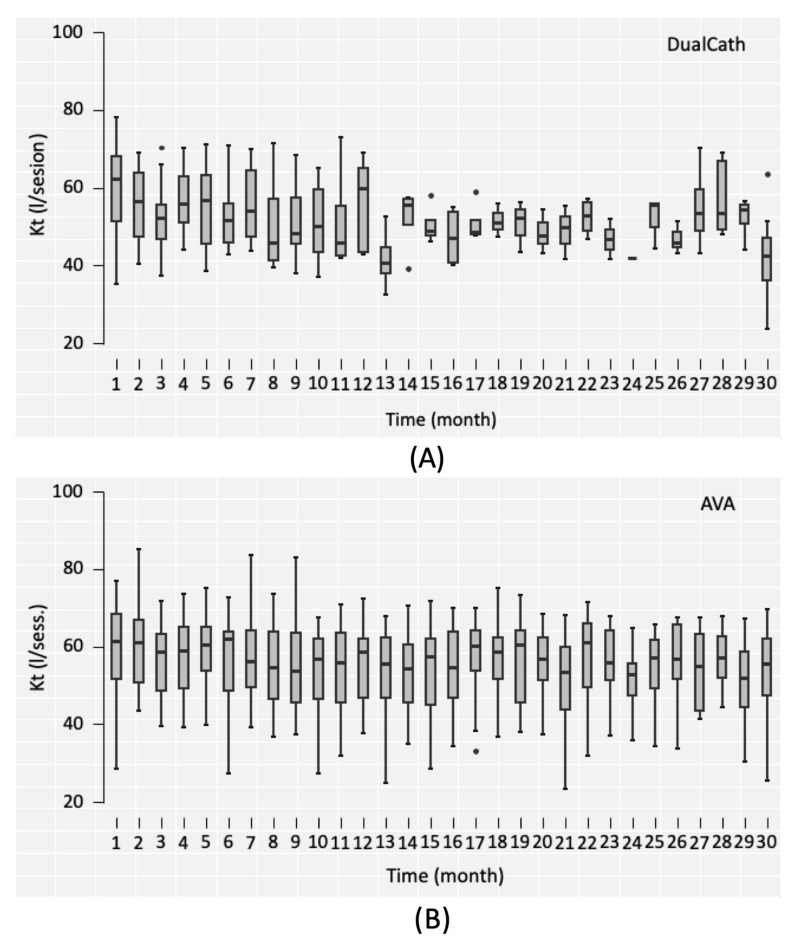
(**A**) The effective urea clearance (eKt) achieved in liters per session over the 30-month follow-up period using DualCath (abbreviated as DCath). (**B**) The effective urea clearance (eKt) achieved in liters per session over the same 30-month follow-up period using arteriovenous access (abbreviated as AVA).

**Figure 4:**
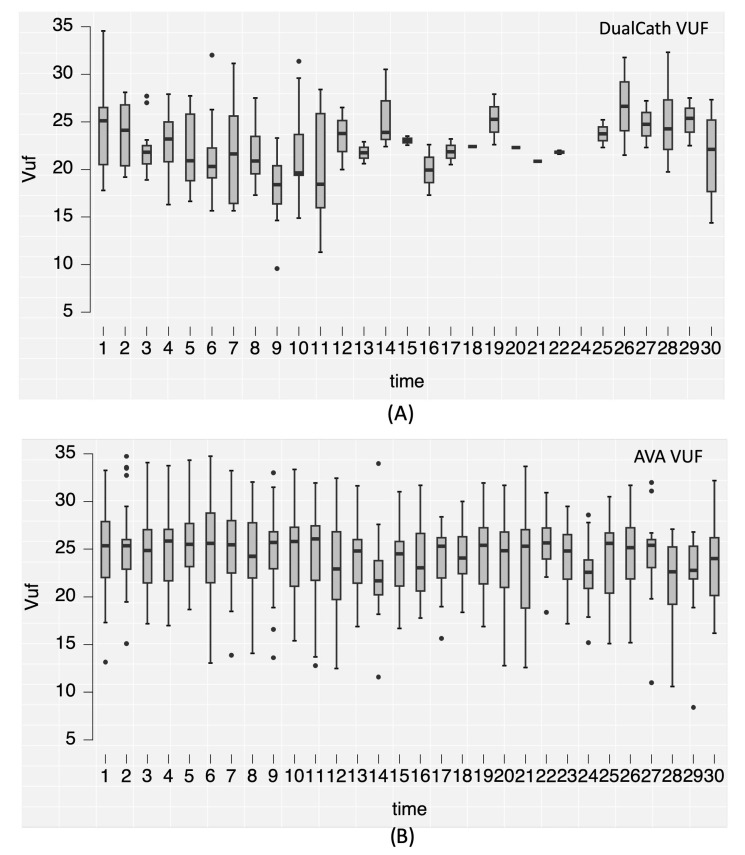
(**A**) The convective volume (Vuf) achieved in liters per session over the 30-month follow-up period using DualCath (abbreviated as DCath). (**B**) The convective volume (Vuf) achieved in liters per session over the same 30-month follow-up period using arteriovenous access (abbreviated as AVA).

Two main indicators of dialysis dose delivery including the effective urea clearance throughout the complete session (Figure 3) and the middle and large molecule clearances as reflected by the total ultrafiltration volume (V_UF_) (Figure 4) were monitored throughout the 30 months of follow-up for the two types of vascular access (AVA, arterio-venous access and DC, DCath). As shown for both cases, the dialysis dose delivered was maintained as relatively stable over the total observational period. The small molecule clearances were slightly higher with AVA than they were with DCath, while the total ultrafiltration volume of AVA—used in this case as a surrogate for middle molecule clearance—was not different from DCath.

**Figure 5:**
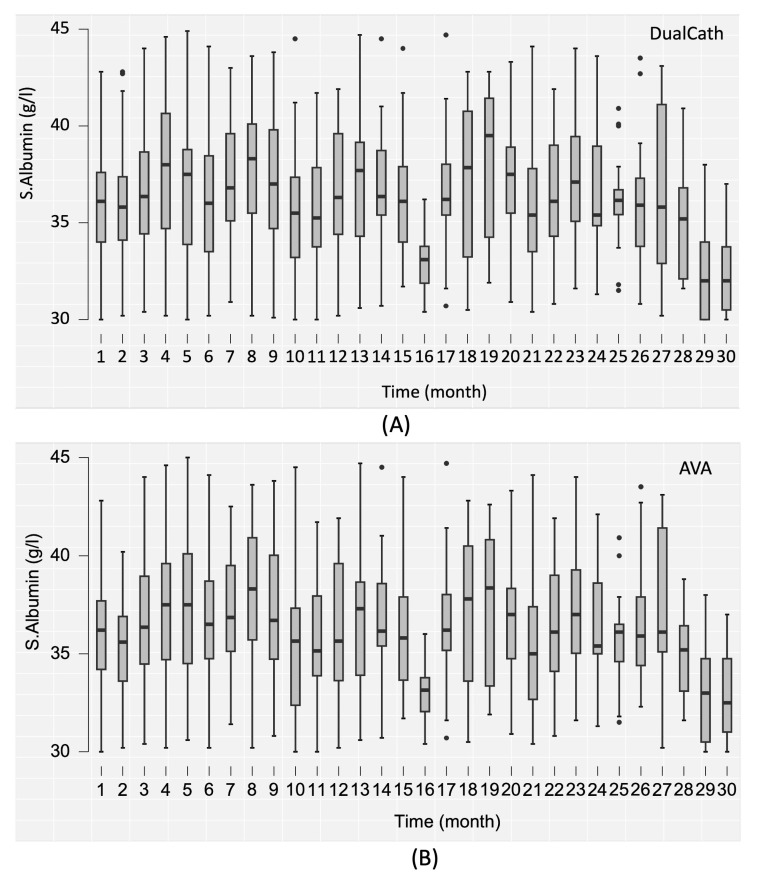
(**A**) The serum albumin (s.albumin) values in grams per liter (g/L) over the 30-month follow-up period using DualCath (abbreviated as DCath). (**B**) The serum albumin (s.albumin) values in grams per liter (g/L) over the same 30-month follow-up period using arteriovenous access (abbreviated as AVA).

The serum albumin concentration—used as an integrated biomarker of dialysis adequacy, safety of the method, and nutritional and inflammation status—was also monitored over the 30 months and is reported in Figure 5.

## 4. Discussion

### 4.1. Main Findings of Our Study

In this retrospective study consisting of 68 maintenance dialysis patients, we precisely assessed the clinical performances delivered throughout tunneled central venous catheters (DCaths) and compared them to the various arteriovenous accesses (AVAs) used routinely in the studied dialysis facility. For this purpose, we used monthly quality control measures with 30 months as the follow-up period. The most original findings of our study were that the DCaths were shown to deliver high blood flow (400 mL/min) and achieve high volume hemodiafiltration on a regular basis, with comparable results to AVAs. As a secondary finding, the study highlighted the fact that the dialysis dose delivered, either for small-sized molecules (total urea clearance) or middle-sized molecules (total ultrafiltration volume and β2M reduction rate), which was almost similar for both DCaths and AVAs, was higher than the targets recommended by clinical best practice guidelines, and it remained stable over time up to 30 months. The main difference was that recirculation with a DCath was significantly higher (≈7%) than that with an AVA, resulting in a total urea clearance when using a DCath that was slightly lower than when using an AVA. Interestingly, this recirculation did not impact the total ultrafiltration volume, which remained similar for both DCaths and AVAs. In all cases, the dialysis performances and key performance indicators were not affected, and DCaths were used as the mainstream vascular access instrument.

### 4.2. Literature Comparison

Our findings contrasted with previous studies reporting that tunneled CVC tends to be associated with a reduction in dialysis dose delivered due to the limited blood flow and increase in vascular access recirculation, but this also precludes the use of high volume hemodiafiltration [21,40,41]. In fact, the apparent superiority of a DCath compared to the other tunneled central venous catheters relied on three main components: firstly, the geometry and the design of the cannulas that were specifically engineered (two independent cannulas, adjustable to patient anthropometry, with side holes at the tips) to provide high blood flow with relatively low blood flow resistance; secondly, the careful way of inserting and positioning the tips in the central venous system at time of implantation, mainly at the junction of the superior vena cava and the right atrium for the internal jugular vein site, and alternatively, in the inferior vena cava for the femoral site; and thirdly, the strict and careful catheter handling protocol consisting of using locking solutions (antithrombotic and fibrinolytic) to maintain or restore catheter flow permeability [2].

Each of these components has been clearly documented in the scientific literature [13]. Two single cannulas made of soft silastic with adequate inner lumens (≥2 mm) and distal side holes tend to provide higher and more sustainable blood flow during a dialysis session than double-lumen semi-rigid polyurethane conventional catheters [12,13,21,42]. The positioning of the tips at the junction of the superior vena cava and the right atrium also tends to reduce risk of poor blood flow occurring during a dialysis session due to the hypovolemia associated with the ultrafiltration [43]. In addition, side holes present at the tips of the catheters keep the lines in the core flow of the vein and prevent sucking vein walls. The higher flow achieved with the side hole catheters has been challenged in some studies by thrombosis and infectious risk [14,15].

Silastic cannulas made of soft rubber tend to be the most hemocompatible material for reducing platelet adhesion, catheter thrombosis risk, and bacterial adhesion [44]. This mechanistic interpretation of the phenomenon has been illustrated in some catheter flow modeling studies, from simulations in silico experiments [45]. Another important factor relies on the strict implementation of best practice catheter-handling developed by nursing teams [46]. Aside from the strict aseptic conditions applied to catheter handling, a new science of catheter locking solutions has been developed, and it has been shown to be very efficient in maintaining catheter lumen patency [46,47,48,49,50]. It is not our intent to review catheter locking practices, but rather, we refer readers interested to recent comprehensive reviews on this topic. In our unit, locking solutions have been always at the heart of catheter care practices. We moved from a conventional heparin locking solution to more personalized and adaptative locking solutions involving more citrate-based solutions and/or fibrinolytic solutions guided by blood flow and/or pressure regime changes in recent times.

### 4.3. Strength and Weakness of Our Study

The strength of our study relies on its precise quantification and comparison of dialysis performances achieved with DCaths compared to the conventional arteriovenous accesses (AVF and AVG) used to deliver high volume hemodiafiltration in a relatively large number of dialysis patients followed up for 30 months. This is in clear contrast with a previous large cohort study showing that high volume HDF (>21 l) was achieved in 33% of prevalent patients [51]. The safety and risk assessment associated with DCath use is currently being assessed in another multicenter study.

Our study had weaknesses that we want to highlight. Firstly, this was a single-center study performed by well-trained physicians and nursing teams dedicated to vascular access management, and therefore, its transferability to larger centers may be questionable. Secondly, the number of patients may appear limited, but this was due to the academic hospital-based dialysis facility with its large turnover of patients. Thirdly, a comparative economic evaluation between DCaths and AVAs was not performed in this study.

### 4.4. Implications for Clinical Practices

As our study indicated, the use of tunneled CVCs is not associated with worse vascular access scenarios in dialysis patients. As such, by means of DCaths, we were able to perform high-volume hemodiafiltration with high blood flows in all patients for sustained periods of up to 30 months. This is a quite reassuring finding since it makes it possible to consider treating fragile patients (i.e., elderly, diabetic, or cardiac patients) or patients without AVF or AVG using the most efficient convective-based treatment modalities, which currently involve high volume hemodiafiltration. In other words, a tunneled central venous catheter should not be considered as a limiting factor in providing the most efficient treatment modality, provided the correct tunneled CVC is used.

## 5. Conclusions

As shown in this study, a high-flow DCath—a tunneled two-single-lumen silicone catheter—may be used in a dialysis-dependent chronic kidney disease patient to deliver high-volume hemodiafiltration, similar to arteriovenous accesses. Although the results obtained with DCaths tended to be slightly lower than those achieved with arteriovenous accesses, they remained far above the best practice guidelines, indicating that the DCaths were not compromising the optimal clinical performances. In addition, the clinical performances of the DCaths remained stable and were achieved in a consistent manner over time. These results support the notion that the design and geometry of the two silicone cannulas were optimal and that the strict adherence to best catheter practices was the key to success for the tunneled central venous catheter use.

## Figures and Tables

**Table 1:** Clinical performance at baseline during the initial 3 months of treatment. The table presents the baseline clinical performances attained during the first three months of treatment. All values are expressed as medians with the corresponding 25th and 75th percentiles.

	AVF	AVG	RFDC	RIJDC	LIJDC	THOMAS S
N patients	36	3	1	25	1	2
n measures	99	4	3	57	3	6
QB (mL/min)						
Median (25–75%)	399 (395–403)	400 (395–403)	400 (395–403)	396 (390–403)	395 (390–403)	400 (400–400)
Venous Presssure (mmHg)						
Median (25–75%)	190 (175–215)	205 (194–218)	265 (244–282)	215 (200–230)	225 (200–230)	130 (120–135)
Recirc VA (%)						
Median (25–75%)	2.64 (1.37–4.80]	2.87 (2.1–3.83)	6.42 (6.18–7.91)	7.01 (4.3–8.62)	13.53 (10.63–15.85)	2.78 (2–3.79)
spKt/V						
Median (25–75%)	1.76 (1.54–1.98)	1.98 (1.94–2.55)	1.95 (1.89–2.00)	1.70 (1.64–1.90)	2.02 (1.98–2.04)	2.18 (2.11–2.24)
dpKt/V						
Median (25–75%)	1.53 (1.31–1.69)	1.72 (1.68–2.14)	1.71 (1.65–1.72)	1.47 (1.30–1.59)	1.71 (1.67–1.74)	1.89 (1.84–1.94)
PR B2M (%)						
Median (25–75%)	76.7 (73.2–79.9)	85.8 (83.5–88.1)	72.6 (70.5–76.5)	NA	75.4 (75.4–75.4)	83.3 (82.8–83.8)
Vsub (L/ses)						
Median (25–75%)	23.3 (20,8–23.3)	24.0 (22.5–24.0)	33.0 (28.7–33)	21.5 (18.0–21.5)	31.5 (25.7–31.5)	29.5 (25.2–29.5)
Vuf (L/ses)						
Median (25–75%)	25.240 (22.0–25.2)	25.7 (24.1–25.7)	35.3 (21.3–35.3)	22.9 (20.5–22.9)	32.1 (29.2–32.1)	31.67 (26.6–31.7)

Abbreviations: AVF, native arteriovenous fistula; AVG, arteriovenous graft PTFE; RFDC, right femoral DualCath; RIJDC, right internal jugular DualCath; LIJDC, left internal jugular DualCath; Qb, blood flow; REC.VA, vascular access recirculation; PR, percent reduction; V_sub_, substitution volume; V_uf_, ultrafiltration volume; spKt/V, single pool Kt/V; dpKt/V, double pool Kt/V.

**Table 2:** Cumulative clinical performances comparing DualCaths (DCaths) and grouped arterio-venous accesses (AVAs) (over a 30-month follow-up period). The median and IQR values are shown for both groups of vascular access.

	AVA	DualCath	*p* Value
N patients	41	27	
n measures	741	207	
BW (Kg)			
median (IQR)	64.8 (20.7)	60.6 (23.4)	0.001
H (%)			
median (IQR)	36.5 (4.5)	36.8 (5.5)	0.716
QB (mL/min)			
median (IQR)	400.3 (15.5)	400.3 (18,4)	0.028
spKt/V			
median (IQR)	1.90 (0.4)	1.80 (0.3)	0.001
eKt/V			
median (IQR)	1.6 (0.32)	1.52 (0.27)	<0.001
REC VA (%)			
median (IQR)	2.8 (3.6)	7.2 (5.2)	<0.001
Kt (L/ses)			
median (IQR)	57 (16.1)	52.6 (16)	0.028
PR-β2M (%)			
median (IQR)	78.0 (8.25)	74.0 (4.0)	0.327
VUF (L/ses,)			
median (IQR)	25.4 (5.5)	22.5 (6.7)	0.098
S-ALB (g/L)			
median (IQR)	35.8 (5.5)	35.6 (6.0)	0.05
PRE-ALB (mg/L)			
median (IQR)	300 (120)	300 (150)	0.437
CRP (mg/L)			
median (IQR)	6.4 (11.Ø)	4.2 (8.8)	0.378
Fib (g/L)			
median (IQR)	4.3 (1.3)	4 (1.5)	0.065
nPCR (g/kg/24 h)			
median (IQR)	0.9 (0.5)	1.0 (0.5)	0.173

Abbreviations: AVA, arteriovenous access; BW, body weight; Ht, hematocrit; Qb, blood flow; REC.VA, vascular access recirculation; PR, percent reduction; V_uf_, total ultrafiltration volume; pre-alb, pre-albumin; nPCR, normalized protein catabolic rate.

## Data Availability

Data are available with restrictions to privacy and ethical constraints.
